# A new artificial diet for western corn rootworm larvae is compatible with and detects resistance to all current Bt toxins

**DOI:** 10.1038/s41598-018-23738-z

**Published:** 2018-03-29

**Authors:** Dalton C. Ludwick, Lisa N. Meihls, Man P. Huynh, Adriano E. Pereira, B. Wade French, Thomas A. Coudron, Bruce E. Hibbard

**Affiliations:** 1 Division of Plant Sciences, University of Missouri, Columbia, Missouri United States of America; 2 Plant Genetics Research Unit, USDA-Agricultural Research Service, Columbia, Missouri United States of America; 3 Department of Plant Protection, Can Tho University, Can Tho, Vietnam; 4 United States Department of Agriculture-Agricultural Research Service, Brookings, South Dakota, United States of America; 5 Biological Control of Insects Research Laboratory, USDA-Agricultural Research Service, Columbia, Missouri United States of America; 6 Present Address: Evogene Inc., BRDG Park at the Danforth Center, 1005 N. Warson Road, Suite 305, St. Louis, Missouri United States of America

## Abstract

Insect resistance to transgenic crops is a growing concern for farmers, regulatory agencies, the seed industry, and researchers. Since 2009, instances of field-evolved Bt resistance or cross resistance have been documented for each of the four Bt proteins available for western corn rootworm (WCR), a major insect pest. To characterize resistance, WCR populations causing unexpected damage to Bt maize are evaluated in plant and/or diet toxicity assays. Currently, it is not possible to make direct comparisons of data from different Bt proteins due to differing proprietary artificial diets. Our group has developed a new, publicly available diet (WCRMO-1) with improved nutrition for WCR larvae. For the current manuscript, we tested the compatibility of all Bt proteins currently marketed for WCR on the WCRMO-1 diet and specific proprietary diets corresponding to each toxin using a susceptible colony of WCR. We also tested WCR colonies selected for resistance to each protein to assess the ability of the diet toxicity assay to detect Bt resistance. The WCRMO-1 diet is compatible with each of the proteins and can differentiate resistant colonies from susceptible colonies for each protein. Our diet allows researchers to monitor resistance without the confounding nutritional differences present between diets.

## Introduction

Western corn rootworm (*Diabrotica virgifera virgifera* LeConte, WCR) has been a challenge for maize (*Zea mays* L.) farmers throughout much of the United States of America (USA) for decades. In 1909, this pest was discovered to attack maize roots in Colorado^1^. Since then, it has expanded its geographic distribution to most of the maize growing regions of North America. Multiple introductions of WCR to Europe^2^ have increased its global importance. More than 30 years ago, it was estimated that the species caused over $1 billion (USD) in economic losses stemming from yield loss and control costs^3^.

Early attempts at managing WCR in the USA focused solely on crop rotation to a non-host such as soybean (*Glycine max* (L.) Merrill) or sorghum^1^. When they became available, management tactics included the application of insecticides for larval or adult management^4,5^. Recently, biotechnology has allowed farmers to grow maize which expresses one or more proteins from *Bacillus thuringiensis* Berliner (Bt). Except for banded applications of soil insecticides, each of these options has failed in one or more regions found within the distribution of this pest^6–14^.

When the first Bt events targeting lepidopterans were registered, insect resistance management (IRM) programs were required. Some believe these programs are especially important for products expressing toxins at less than high-dose or species with lesser susceptibility to the proteins^15^, since the likelihood of survival increases when products express toxins at less than high-dose, or when some targeted species are less susceptible to the toxins. Recently, biotechnology has allowed farmers to grow maize that expresses one or more proteins from *Bacillus thuringiensis* Berliner (Bt) for WCR control. However, modeling efforts and laboratory selection studies for current proteins targeting WCR suggested products would lose efficacy within a decade of first use^16–21^, and field data support this^10,13,14^. Bt maize products targeting WCR have not yet met the high-dose criterion^22^, and this likely is a primary reason that the refuge strategy designed to delay resistance has been ineffective with this pest^23^.

Resistance monitoring efforts to determine whether a shift in the susceptibility of randomly sampled populations was required as part of the registration process and to date has used proprietary artificial diets developed by product registrants^24–26^. While this effort meets the requirement for IRM programs, there have been issues with the diet toxicity assays. In addition to nutritional differences between diets, high levels of contamination often occurred. Changes in assay methodology and diet formulations have also occurred over the years for some Bt proteins, likely creating additional variability in the data. Some academic researchers have tended to use on-plant assays as opposed to proprietary diets and purified Bt proteins, which require special agreements with the owners. This barrier can be overcome with relationships between industry and academia, but often takes considerable time and negotiation. As a result, several studies have evaluated Bt resistance with on-plant assays only^10–12,21,27–29^, and some with both on-plant and diet toxicity assays^13,14,17,19,30–32^. Following initial reports of field-evolved Bt resistance using on-plant assays, the Environmental Protection Agency (EPA) proposed on-plant assays be used as a replacement for diet toxicity assays^33^ and the proposed changes for resistance monitoring have been implemented^34^. Instead of diet toxicity assays, registrants will be required to use on-plant assays to determine shifts in susceptibility, though they may continue to conduct diet toxicity assays in conjunction with on-plant assays.

Efforts have been underway to improve WCR diets and the first new publicly available diet was recently published^35^. Referred hereafter as “WCRMO-1”, this diet has improved characteristics compared to the only other publicly-available diet^36^ and all the proprietary diets used in the current study (Hibbard/Coudron labs unpublished data). Efforts are continuing to further improve the WCRMO-1 diet for weight gain and developmental rate. An optimum diet for WCR should match maize for survival, weight, and developmental rate parameters. If these parameters can be optimized, or can no longer be improved, then we believe a single diet should be used as a universal diet in all public diet toxicity assays. As a step toward this longer-term goal, we compared the WCRMO-1 diet to the appropriate proprietary diets for compatibility with Bt proteins. We also tested susceptible and resistant WCR colonies on the WCRMO-1 diet to evaluate the ability of the diet to detect differences in susceptibility to Bt proteins in these two phenotypes.

## Results

### Lethal effects

**Figure 1:**
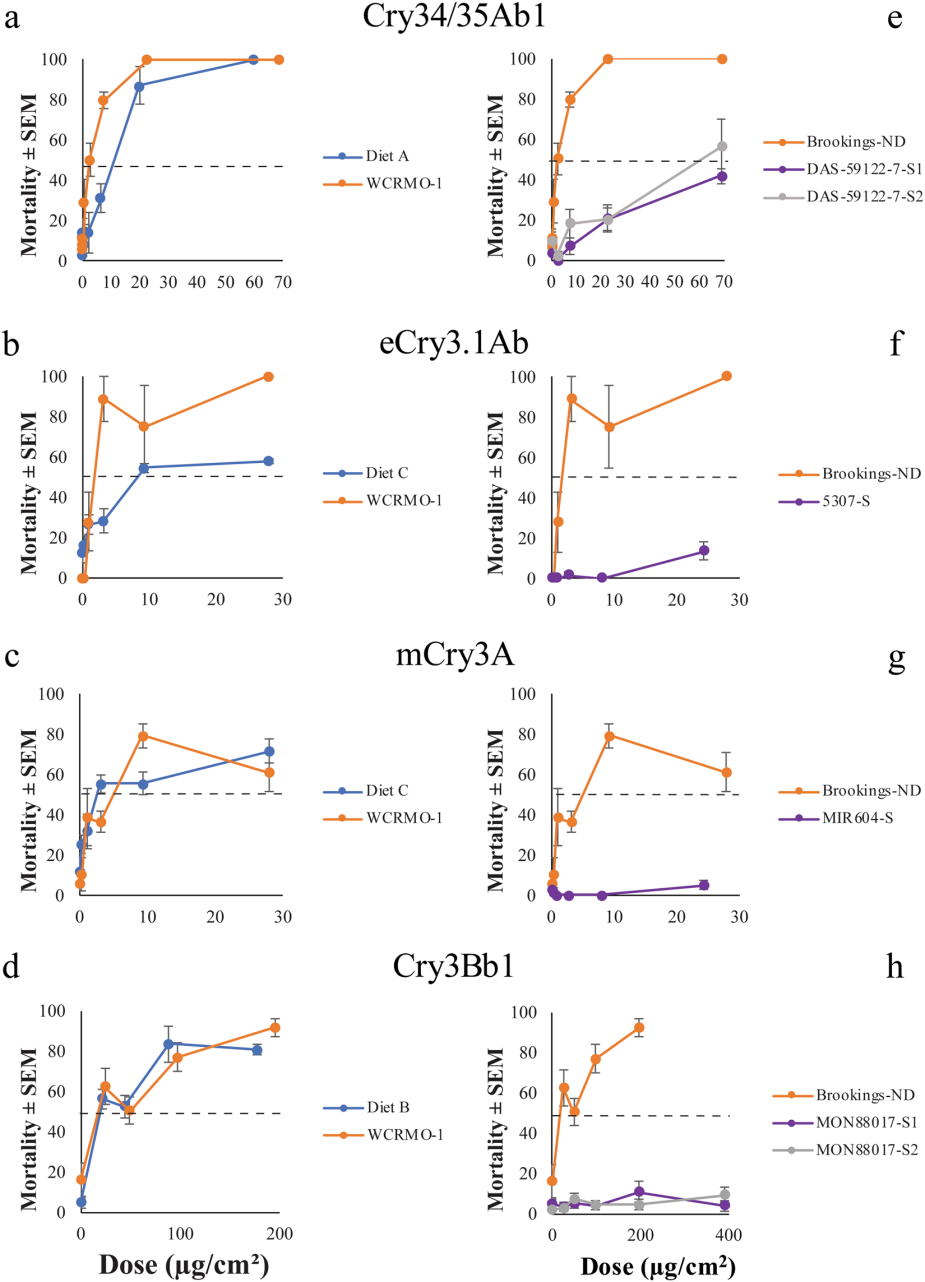
Impact of Bt Protein on Mortality. Percent mortality of Brookings-ND (**a**–**d**) with exposure to Bt proteins on proprietary and WCRMO-1 diets, and mortality of Brookings-ND and selected colonies (**e**–**h**) with exposure to Bt proteins on the WCRMO-1 diet. Mortality was calculated as the number of larvae that died following exposure to Bt protein(s) divided by the initial number infested. Bars represent the standard error of the mean (SEM). The dashed line represents 50 percent mortality.

The WCRMO-1 diet was tested with each Bt protein alongside the proprietary diet of the corresponding registrant using a Bt-susceptible colony (Brookings-ND, Table 1). Average mortality for the buffer dose (control) on WCRMO-1 was less than 10 percent for all but one instance (Fig. 1d). The average mortality for the buffer dose on proprietary diet C was between 12 and 13 percent (Fig. 1b and c), while the buffer dose for proprietary diets corresponding to Cry34/35Ab1 (proprietary diet A) and Cry3Bb1 (proprietary diet B) toxins was less than five percent (Fig. 1a and d). Based on overlapping confidence intervals between concentrations required to kill 50 percent of insects (LC_50_), WCRMO-1 provided similar data as the proprietary diet for each protein except for one (Table 2). WCRMO-1 produced a significantly lower LC_50_ value for eCry3.1Ab compared to the corresponding proprietary diet, labelled as “proprietary diet C” (Table 2). This means less eCry3.1Ab was required on WCRMO-1 to kill half of the susceptible colony tested when compared to proprietary diet C.

**Table 1:** Buffers, doses, and proteins used with each colony.

Protein	Diet	Colony	Protein Buffer	Dose (µg/cm^2^)							
				Dose 1	Dose 2	Dose 3	Dose 4	Dose 5	Dose 6	Dose 7	Dose 8
Cry34/35Ab1	Proprietary Diet A	Brookings-ND	10 mM Sodium Citrate pH 3.5	0.00	0.08	0.24	0.74	2.22	6.67	20.00	60.00
	WCRMO-1	Brookings-ND		0.00	0.09	0.28	0.85	2.55	7.65	22.94	68.82
		DAS-59122-7-S1 (6 gen.)		0.00	0.09	0.28	0.85	2.55	7.65	22.94	68.82
		DAS-59122-7-S2 (18 gen.)		0.00	0.09	0.28	0.85	2.55	7.65	22.94	68.82
Cry3Bb1	Proprietary Diet B	Brookings-ND	10 mM Sodium Carbonate Bicarbonate pH 10	0.00	22.19	44.29	88.57	177.14	.	.	.
	WCRMO-1	Brookings-ND		0.00	24.54	48.97	97.95	195.89	.	.	.
		MON88017-S1 (11 gen.)		0.00	24.54	48.97	97.95	195.89	391.79	.	.
		MON88017-S2 (11 gen.)		0.00	24.54	48.97	97.95	195.89	391.79	.	.
mCry3A	Proprietary Diet C	Brookings-ND		0.00	0.34	1.03	3.10	9.29	27.87	.	.
	WCRMO-1	Brookings-ND		0.00	0.34	1.03	3.10	9.29	27.87	.	.
		MIR604-S (50 + gen.)		0.00	0.30	0.90	2.70	8.10	24.30	.	.
eCry3.1Ab	Proprietary Diet C	Brookings-ND		0.00	0.34	1.03	3.10	9.29	27.87	.	.
	WCRMO-1	Brookings-ND		0.00	0.34	1.03	3.10	9.29	27.87	.	.
		5307-S (35 gen.)		0.00	0.30	0.90	2.70	8.10	24.30	.	.

**Table 2:** Concentrations (µg/cm^2^) to kill 50 percent (LC_50_), cause 50 percent weight inhibition (EC_50_ values), and cause 50 percent molt inhibition (MIC_50_) with 95 percent confidence intervals for corresponding colony, diet, and Bt protein treatments.

Bt Protein	Diet	Colony	Reps	LC_50_ (95% C.I.)	EC_50_ (95% C.I.)	MIC_50_ (95% C.I.)
Cry34/35Ab1	Proprietary Diet A	Brookings-ND	3	6.28 (2.60–22.64)	2.01 (0.33–12.05)	0.96 (0.33–2.85)
	WCRMO-1	Brookings-ND	4	1.60 (0.83–3.15)	1.31 (0.15–11.26)	0.92 (0.27–3.35)
		DAS-59122-7-S1 (6 gen.)	5	>68.82	5.99 (3.37–9.13)	3.48 (0.59–18.51)
		DAS-59122-7-S2 (18 gen.)	4	>68.82	3.09 (1.38–4.94)	10.96 (4.4–25.3)
Cry3Bb1	Proprietary Diet B	Brookings-ND	4	9.11 (2.15–24.69)	12.41 (1.00–87.62)	2.19
	WCRMO-1	Brookings-ND	3	3.36 (0.35–15.17)	10.20 (0.32–88.80)	0.48
		MON88017-S1 (11 gen.)	5	>391.8	409.23 (250.35–1092.77)	>391.8
		MON88017-S2 (11 gen.)	4	>391.8	614.77 (333.64–2311.32)	>391.8
eCry3.1Ab	Proprietary Diet C	Brookings-ND	3	12.74 (5.10–61.54)	4.02 (1.89–9.20)	N/A
	WCRMO-1	Brookings-ND	4	1.75 (0.93–3.57)	20.09	0.35 (0.02–2.44)
		MIR604-S (35 gen.)	5	>24.3	>24.3	>24.3
mCry3A	Proprietary Diet C	Brookings-ND	5	3.71 (1.93–8.08)	3.13 (1.80–5.42)	N/A
	WCRMO-1	Brookings-ND	6	6.39 (2.82–20.39)	1.23 (0.14–9.7)	0.5 (0.12–1.48)
		MIR604-S (50 + gen.)	5	>24.3	>24.3	>24.3

Non-overlapping confidence intervals indicate significant differences. Confidence intervals could not be calculated for some of the estimates.

When Bt-selected colonies were evaluated on WCRMO-1, significant differences in LC_50_ values were found for each Bt protein between the Brookings-ND colony and the resistant colonies (Table 2). This was the case for each selected colony on each of the four Bt toxins evaluated. Overall, resistant colonies showed minimal mortality in response to toxins. For Cry3Bb1, mCry3A, and eCry3.1, all resistant colonies had maximum mortality under 14 percent regardless of the dose (Fig. 1f–h). Only one of the DAS-59122-selected colonies reached maximum mortality 50% while the other was 41% following exposure to the highest dose Cry34/35Ab1 toxins (Fig. 1e).

### Sublethal effects

Dry weight was the only parameter where a significant difference was observed between susceptible larvae fed Bt toxin on WCRMO-1 versus a proprietary diet. For eCry3.1Ab, the concentration required to cause a 50 percent reduction in dry weight (EC_50_) for the Brookings-ND colony fed WCRMO-1 diet was significantly greater than when fed proprietary diet C (Table 2). No other significant differences were found in EC_50_ values between the WCRMO-1 diet and the respective proprietary diet for any of the other toxins (Table 2). Both DAS-59122-7-selected colonies had similar EC_50_ values compared to the Brookings-ND colony when exposed to the Cry34/35Ab1 proteins on the WCRMO-1 diet (Table 2). Both MON88017-selected colonies had significantly greater EC_50_ values for Cry3Bb1 protein on the WCRMO-1 when compared to the susceptible colony (Table 2). The Brookings-ND colony had a significantly lower EC_50_ value for eCry3.1Ab protein on the WCRMO-1 diet compared to the 5307-S colony (Table 2). Lastly, the MIR604-S colony had a significantly greater EC_50_ value for mCry3A protein when compared to the susceptible colony on the WCRMO-1 diet (Table 2).

**Figure 2:**
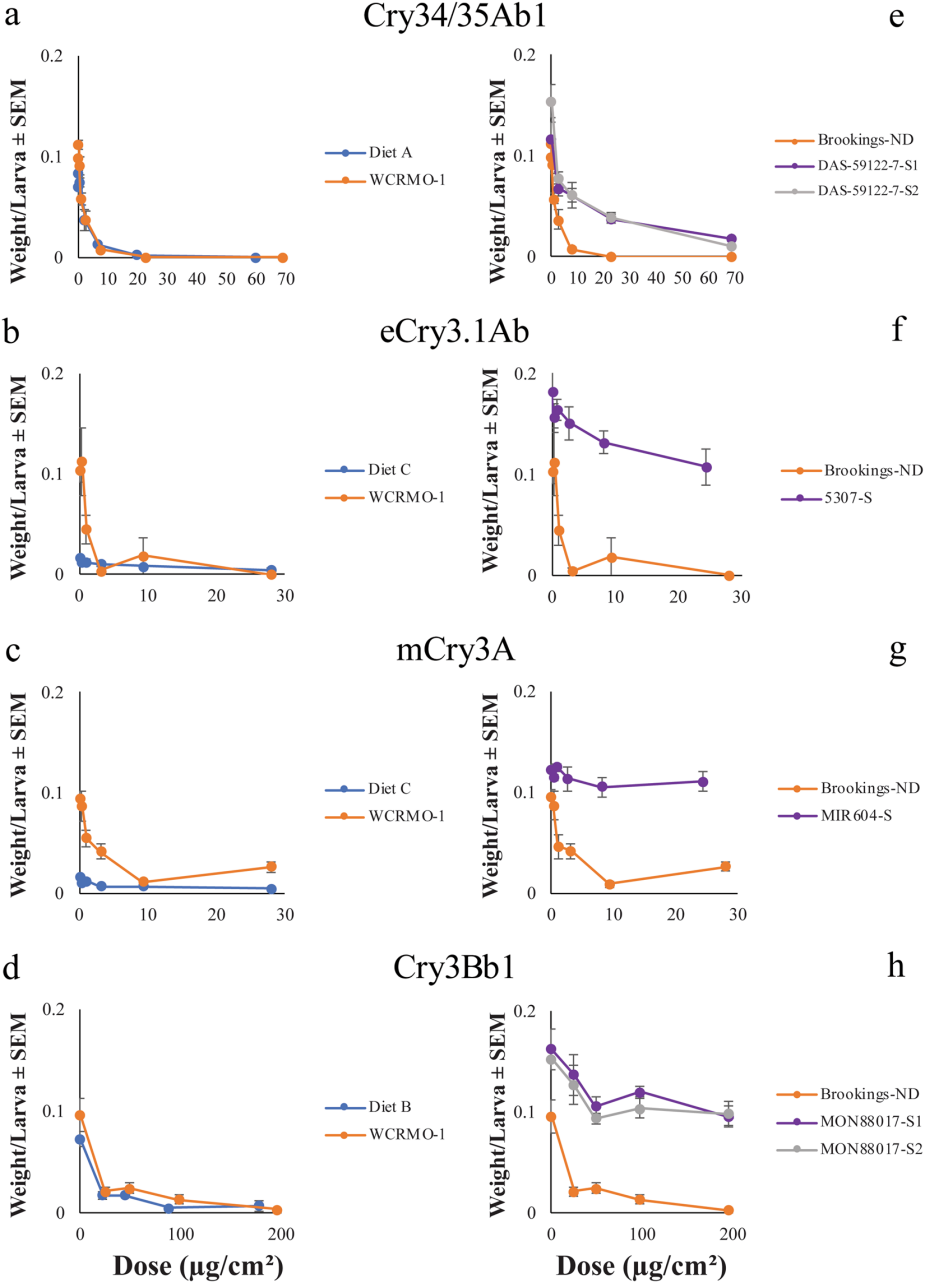
Impact of Bt Protein on Dry Weight. Dry weight per larva (mg) of Brookings-ND (**a**–**d**) with exposure to Bt proteins on proprietary and WCRMO-1 diets, and dry weight per larva of Brookings-ND and selected colonies (**e**–**h**) with exposure to Bt proteins on the WCRMO-1 diet. Dry weight per larva was calculated as the dry weight of larvae recovered following exposure to Bt protein(s) divided by the initial number infested. Bars represent the standard error of the mean (SEM).

There were large differences in susceptible WCR larval dry weight on the buffer dose between the WCRMO-1 and proprietary diet C (Fig. 2b and c). For both toxins, the insects reared on proprietary diet C weighed one-fifth as much as insects reared on WCRMO-1 after 10 d (Fig. 2b and c). Insects from Brookings-ND exposed to the buffer dose of Cry34/35Ab1 weighed more on WCRMO-1 than those reared on proprietary diet A. All but one colony (DAS-59122-7-S1) had greater dry weight than the Brookings-ND insects on the buffer dose (Fig. 2e–h). Additionally, some colonies had greater dry weight at the lowest Bt dose than for the buffer (Fig. 2a,d–f).

**Figure 3:**
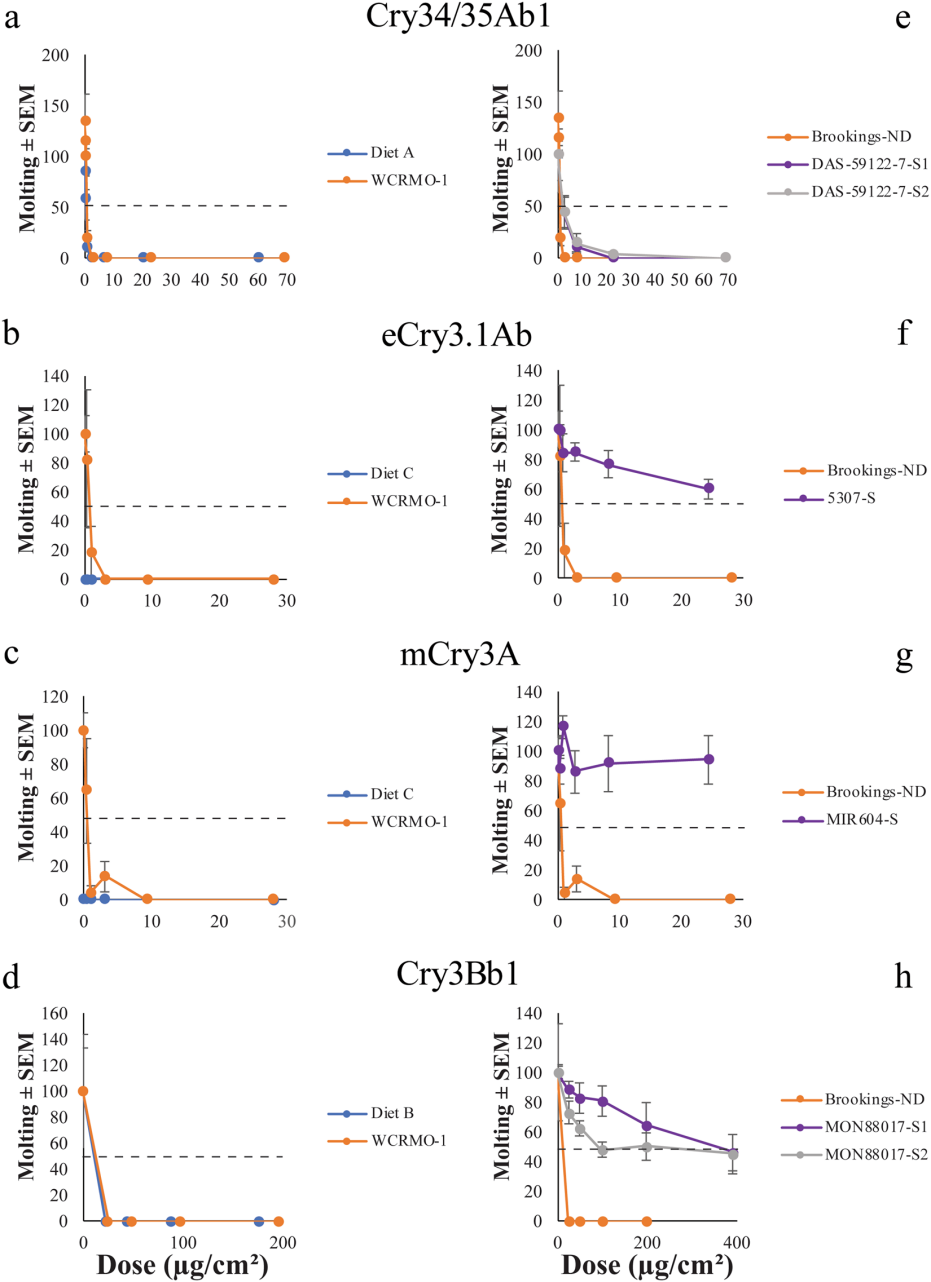
Impact of Bt Protein on Molting. Molting of Brookings-ND (**a**–**d**) with exposure to Bt proteins on proprietary and WCRMO-1 diets, and molting of Brookings-ND and selected colonies (**e**–**h**) with exposure to Bt proteins on the WCRMO-1 diet. First, the molting rate on the buffer was calculated by dividing the number of larvae which molted by the initial number of larvae. This established a baseline response of the insect to the diet and buffer. All values for each colony, including the buffer dose (Dose 1) for the same colony, were then divided by the buffer dose value to establish relationships relative to the buffer dose. Each of the resulting values were then multiplied by 100. Bars represent the standard error of the mean (SEM).

Molting was significantly inhibited for all susceptible colonies on all Bt proteins (Fig. 3a–d). The dose that causes 50 percent molting inhibition (MIC_50_) was significantly greater for all the Cry3-selected colonies than the Brookings-ND colony (Table 2). The MIC_50_ value was not significantly different for the two Cry34/35Ab1-selected colonies and the Brookings-ND colony (Table 2).

## Discussion

Here, we demonstrate for the first time that a single artificial diet can be used to detect resistance with all Bt proteins currently targeting WCR. Previously, different proprietary proteins were evaluated on the proprietary diet from the company that produced the protein. Use of a single diet allows for direct comparisons of toxicity between proteins without the confounding effects of nutritional differences. In general, performance of susceptible WCR was similar between the proprietary diets and the WCRMO-1 diet (Table 2, Figs 1–3). We also documented that differences between WCR colonies selected for resistance to each Bt protein and a control WCR population can be detected in diet toxicity assays with the WCRMO-1 diet (Table 2, Figs 1–3).

This study adds to the limited number of studies which have documented a significant difference in LC_50_ values between colonies or populations in Cry34/35Ab1 diet toxicity assays^13,14^. Resistance ratios in the previous studies were relatively minor compared to the more than 40 fold difference in LC_50_ values of the two DAS-59122-7-selected populations and the control population evaluated here (Table 2). Interestingly, a colony originally described by Lefko *et al*.^30^ and Nowatzki *et al*.^37^ showed no difference in LC_50_ values when compared to a control colony after being selected for more than 30 generations on DAS-59122-7 maize (which produces the Cry34/35Ab1 toxin) using proprietary diet A (pers. communication from Stephen Thompson, DuPont Pioneer). Using the WCRMO-1 diet, significant differences in LC_50_ values between both DAS-59122-7-selected colonies and control colonies were documented with less than 20 generations of selection (Table 2, Figs 1–3). The difference in LC_50_ values did not translate to significant differences in EC_50_ values; however, the DAS-59122-7-S2 colony did have a significantly greater MIC_50_ value than the control WCR colony (Table 2). These results suggest a possibility for more sensitive screening of Cry34/35Ab1 proteins with WCRMO-1. However, there is also a chance that the difference in LC_50_ values detected on the WCRMO-1 diet is unique to the DAS-59122-7-selected colonies in this study. Additional comparisons of proprietary diet A and WCRMO-1 diets are needed to determine whether the basis of this detection is the result of nutritional or genetic factors, or a combination of the two factors.

Although we do not have the formulations for the proprietary diets tested here, life history parameters indicate that nutritive qualities varied among the diets. We previously found significant differences in molting rate, weight gain, and survival among the proprietary and WCRMO-1 diets (unpublished data). Research with lepidopteran insects has shown that the toxicity of Bt proteins can vary when protein to carbohydrate ratios are manipulated^38,39^. While lepidopteran and coleopteran insects are quite different, we may be able to draw parallels to this study. One proprietary artificial diet appears to lack significant nutritive qualities and/or attractiveness as a food source. Insects on proprietary diet C with buffer alone for eCry3.1Ab and mCry3A proteins weighed less than 18 percent of insects reared on the WCRMO-1 diet with buffer alone (Fig. 2b and c). Such a difference in nutritive qualities could explain the significant difference in dry weight observed between larvae fed WCRMO-1 or the proprietary diet C. However, this difference in weight gain between diets did not cause a significant difference in LC_50_ for the mCry3A protein. Under conditions where an insect ingests food at a normal rate (i.e. rate on maize), then the LC_50_ is representative of the amount needed to kill targeted insects in the field. However, when a diet is less attractive, then the LC_50_ will likely shift towards a greater value as less diet is ingested.

Larvae were capable of molting in the 10-day time period used for these experiments, unlike previously reported assays with WCR which were terminated at five or six days depending on the protein. Previously reported assays had shorter time frames primarily due to contamination (EPA 2016). Longer assays for slower acting products (e.g. dsRNA) are possible using techniques described here and in Huynh *et al*.^35^. Consequently, we were able use molting as a measure of resistance, similar to studies with *Ostrinia nubilalis* (Hübner)^40^. Here, exuviae were clearly visible through the sealing film and provided a non-invasive determination of sublethal effects that could be recorded over time, whereas dry weight could only be collected at the end of each experiment. Recording molting data as a measure of resistance may allow for additional studies where researchers may look at genetic factors, microbes, or other variables. This approach may provide a more sensitive measure for detecting resistance in populations where no differences in LC_50_ or EC_50_ exist. For example, the DAS-59122-7-S2 colony had a significantly greater MIC_50_ value when compared to the Brookings-ND colony while the DAS-59122-7-S1 showed no significant difference (Table 2). This was the only difference noted between the two DAS-59122-7 selected colonies. Future studies should include these data, when possible. Before molting data can be collected, a diet must have adequate nutrition to allow first instar larvae to molt into the second instar. Proprietary diet C did not produce second instar larvae, even on diet with buffer alone (Fig. 3b and c). All other diets produced second instar larvae on diet with buffer alone (Fig. 3a and d).

Nutritional improvements of an artificial diet^35^ and validation of compatibility with all current Bt proteins documented here, support the utility of the diet toxicity assay in resistance monitoring efforts. We recommend a standardized approach to how these diet toxicity assays are conducted to reduce data variability in IRM programs. Some of this variability is likely due to the alterations of some diets over the years for various reasons. Additionally, the number of days for observation varies between proteins complicating comparisons of Bt proteins. These factors likely explain some of the variability that has been reported within diet toxicity assays over the years. Shifts in susceptibility may be masked by these factors. Comparisons between current and future products could help to evaluate novel modes of action and physiological effects of protein exposure.

WCR has evaded management tactics for more than a century. Some populations of this species have developed resistance to crop rotation^9^, broadcast insecticides^6–8^, and transgenic maize expressing Bt proteins^10,11,13,14,27,29^. Even with the advent of biotechnology, WCR continues to be a pest of maize. Current and future products targeting the species will require IRM programs to ensure product viability and longevity. Both plant and diet assays have value in detecting resistance^13^.

## Materials and Methods

### Artificial Diet and Bt Protein

Lyophilized Cry34/35Ab1 proteins and the corresponding proprietary diet were provided by Dow AgroSciences. Cry3Bb1 protein was provided in solution by Monsanto Company in addition to their proprietary diet. The lyophilized mCry3A and eCry3.1Ab proteins and their proprietary diet were provided by Syngenta Biotechnology. The WCRMO-1 diet was produced by the USDA-ARS Biological Control of Insects Research Laboratory (BCIRL) in Columbia, MO^35^. Buffers to suspend the protein in solution were prepared at BCIRL (Table 1). The sodium citrate buffer was stored at 4 °C immediately after preparation. The sodium carbonate bicarbonate buffer was stored at room temperature in a container which blocked all light.

### Insects

Eggs from the non-diapausing, susceptible colony, hereafter known as Brookings-ND, were provided by the USDA-ARS laboratory in Brookings, SD. This colony is derived from a field population collected prior to the release of transgenic crops^41^, so this colony should be susceptible to all Bt proteins active against WCR.

Several resistant colonies were used in this study, all of which have been described in other studies. Two MON88017-selected colonies, MON88017-S1 and MON88017-S2, exhibited resistance to Cry3Bb1-expressing plants (event MON88017) and protein in Zukoff *et al*.^13^. MON88017-S1 was derived from the Canby population while MON88017-S2 was derived from Hills population in Zukoff *et al*.^13^. Both MON88017-selected colonies were reciprocally crossed with adults from the Brookings-ND colony and then reared on maize containing event MON88017 for eleven generations post Zukoff *et al*.^13^ at the time Cry3Bb1 diet toxicity assays were conducted.

Two DAS-59122-7-selected colonies, hereafter known as DAS-59122-7-S1 and DAS-59122-7-S2, were also used in this study. The DAS-59122-7-S1 colony was derived from a field population described in Ludwick *et al*.^14^. The field population had been exposed to SmartStax, a maize product which expresses both Cry34/35Ab1 (event DAS-59122-7) and Cry3Bb1 proteins. DAS-59122-7-S1 had incomplete resistance to both Cry3Bb1 and Cry34/35Ab1 proteins^14^. Eggs from this population were reared on non-Bt corn and resulting adults were then reciprocally crossed with the Brookings-ND colony to produce a non-diapausing DAS-59122-7-S1 colony. The DAS-59122-7-S2 colony was started from pooled eggs of three field populations reared on non-Bt maize. The resulting adults were reciprocally crossed with Brookings-ND at differing points earlier and then selected on maize containing event DAS-59122-7 similarly as in Deitloff *et al*.^21^. The DAS-59122-7-S1 and DAS-59122-7-S2 colonies were selected on DAS-59122-7 plants for six and 18 generations under laboratory/greenhouse conditions, respectively, at the time Cry34/35Ab1 diet toxicity assays occurred.

Two additional colonies with resistance to mCry3A^31^ and eCry3.1Ab^19^ were also tested. The colony selected on eCry3.1Ab-expressing corn will hereafter be known as 5307-S. We tested this strain after 35 generations of selection on maize containing event 5307 or an earlier event also expressing eCry3.1Ab. The MIR604-selected colony will hereafter be known as MIR604-S. The MIR604-S colony was evaluated after more than 50 generations of selection on MIR604 maize which expresses mCry3A protein.

### Equipment Sterilization

The WCRMO-1 diet was prepared in new 96-well immunoassay plates (product #3370, Corning Inc., Corning, NY) using sterile techniques described in Huynh *et al*.^35^ All proprietary diets were prepared in 96-well immunoassay plates by the registrants. For shipment, proprietary diets were placed in sterile packaging and shipped to the BCIRL in styrofoam containers with ice packs. Diets were stored in a 4 °C refrigerator and infested within two weeks of arrival. Lids for diet were only removed inside a UV-sterilized biosafety cabinet. Paintbrushes, insect pins, deli containers, coffee filters, and beakers were all sterilized by UV lights in a biosafety cabinet prior to use. A spray bottle with autoclaved DI water was used to keep the coffee filter and neonate larvae moist.

### Egg Sterilization

The methods used to surface sterilize eggs were modified from descriptions by Pleau *et al*.^36^. Briefly, eggs from each colony were incubated in Petri dishes with 70 mesh sieved soil within an incubator at 25 °C in complete darkness until hatch started. Once approximately 10 percent of the eggs hatched, each dish was washed through a 60 mesh sieve. Remaining eggs were submerged in a beaker with water where debris (soil, fungus, hatched egg shells) floated to the water’s surface and were then decanted. As much excess water as possible was poured off before the eggs were submerged in 20 mL of disinfectant (undiluted Lysol^®^) for three minutes. The disinfectant was then decanted and the eggs were triple rinsed with distilled water. Approximately 20 mL of 10 percent buffered zinc formalin (10 percent formaldehyde) covered the eggs for three minutes. Again, a triple rinse with distilled water was used to remove any chemical residues. Eggs were then dispensed onto a UV-sterilized coffee filter with a 1.5 ml transfer pipette. The coffee filter was then placed in a UV-sterilized 16 oz. Solo^®^ deli container with holes (#0 insect pin) punched in the lid to allow for air exchange. Incubation, typically less than three days, occurred until enough eggs hatched for infestation. If sufficient numbers of larvae did not hatch, then the coffee filter with eggs were transferred to a recently UV-sterilized deli container each additional day and placed back in the incubator. Contamination tends to increase when eggs are used more than three days after sterilization (personal observation). The deli container with larvae on its wall was placed in a sterilized biosafety cabinet. Neonate larvae were then transferred to diet using sterile equipment to prevent any possible contamination. Sealing film (Excel scientific, Inc., Thermalseal RTS^RM^, TSS-RTQ-100) was placed over the entire plate to prevent escape. A single hole (#0 insect pin) was poked into the sealing film of each infested well for diffusion of oxygen.

### Diet Toxicity Assays

All diet toxicity assays were conducted for a length of 10 days. Lyophilized proteins were dissolved in solution and then a serial dilution was made (Table 1). These solutions were then overlaid on artificial diet and allowed to dry before larvae were placed on the diet. Each row (12 wells) per plate received one dose and one colony, thereby constituting a single replication. Plates with more than 25 percent mortality on the control (buffer dose, 0 µg/cm^2^) were excluded from the study. The number of replications included in this study varied between diets and proteins (Table 2). Survival and molting were recorded prior to the collection of larvae. Surviving larvae were placed in ethanol for each dose and plate and then dried in an oven at 50 °C for one week (Blue M Therm Dry Bacteriological Incubator, Model #602752). After drying, larvae were weighed on a digital microbalance (Sartorius^TM^ Cubis^TM^, 6.6S), which weighed samples to one thousandth of a milligram.

### Statistical Analyses

Mortality was calculated by dividing the number of dead larvae by the initial number of larvae infested per dose. An average mortality value of the buffer dose was calculated. Then, mortality at each subsequent dose was divided by the average mortality of the buffer dose and multiplied by 100^42^. These mortality percentages were analyzed with a probit analysis to generate LC_50_ and 95 percent confidence intervals using SAS software version 9.2 (SAS Institute). Values were considered significantly different when 95 percent confidence intervals did not overlap.

Dry weight was calculated by dividing the total weight per dose by the initial number of larvae infested, effectively giving all dead larvae a weight of 0. The average dry weight per larva recovered from the buffer dose was then averaged across replications to generate a mean value. The dry weight of larvae recovered from each dose was then divided by the buffer’s mean dry weight and multiplied by 100 to generate a percentage relative to the buffer dose. These data were then analyzed with a nonlinear probit analysis described by Marçon *et al*.^43^ in SAS. This analysis calculated the EC_50_ and 95 percent confidence intervals.

Molting was calculated by dividing the number of molted individuals per dose by the initial number of larvae infested and multiplying by 100 to obtain a percentage. The average molting rate for each colony on the buffer dose was calculated. Each subsequent dose was divided by the buffer dose’s average molting rate for the corresponding colony^42^. These molting rates were then analyzed in SAS with a probit analysis to generate MIC_50_ values and 95 percent confidence intervals. MIC_50_ values between colonies on the same protein were considered significantly different when confidence intervals did not overlap.

### Data Availability

All pertinent data are found in the figures and tables. Requests for data and additional information should be submitted to the corresponding author.
