# Author Correction: A new artificial diet for western corn rootworm larvae is compatible with and detects resistance to all current Bt toxins

**DOI:** 10.1038/s41598-018-32259-8

**Published:** 2018-09-20

**Authors:** Dalton C. Ludwick, Lisa N. Meihls, Man P. Huynh, Adriano E. Pereira, B. Wade French, Thomas A. Coudron, Bruce E. Hibbard

**Affiliations:** 1 Division of Plant Sciences, University of Missouri, Columbia, Missouri United States of America; 2 Plant Genetics Research Unit, USDA-Agricultural Research Service, Columbia, Missouri United States of America; 3 Department of Plant Protection, Can Tho University, Can Tho, Vietnam; 4 United States Department of Agriculture-Agricultural Research Service, Brookings, South Dakota United States of America; 5 Biological Control of Insects Research Laboratory, USDA-Agricultural Research Service, Columbia, Missouri United States of America; 6 Present Address: Evogene Inc., BRDG Park at the Danforth Center, 1005 N. Warson Road, Suite 305, St. Louis, Missouri United States of America

Correction to: *Scientific Reports* 10.1038/s41598-018-23738-z, published online 29 March 2018

The Article contains an error in Table 2 where the resistant colony tested on WCRMO-1 diet with eCRY3.1Ab protein is incorrectly given as“MIR604-S (35 gen.)”.

The correct colony should read “5307-S (35 gen.)”.

